# Recyclable Carbon Cloth-Supported ZnO@Ag_3_PO_4_ Core–Shell Structure for Photocatalytic Degradation of Organic Dye

**DOI:** 10.3390/toxics11010070

**Published:** 2023-01-11

**Authors:** Yuan Yi, Qifang Guan, Wenguang Wang, Siyuan Jian, Hengchao Li, Liangpeng Wu, Haiyan Zhang, Chuanjia Jiang

**Affiliations:** 1 School of Materials and Energy, Guangdong University of Technology, Guangzhou Higher Education Mega Center 100#, Guangzhou 510006, China; 2 Advanced Energy Science and Technology Guangdong Laboratory, Huizhou 516000, China; 3 College of Environmental Science and Engineering, Nankai University, 38 Tongyan Rd., Tianjin 300350, China

**Keywords:** carbon cloth, silver phosphate, zinc oxide, S-scheme heterojunction, wastewater treatment

## Abstract

The extensive use of organic dyes in industry has caused serious environmental problems, and photocatalysis is a potential solution to water pollution by organic dyes. The practical application of powdery photocatalysts is usually limited by the rapid recombination of charge carriers and difficulty in recycling. In this study, recyclable carbon cloth-supported ZnO@Ag_3_PO_4_ composite with a core–shell structure was successfully prepared by solvothermal treatment and subsequent impregnation–deposition. The as-prepared carbon cloth-supported ZnO@Ag_3_PO_4_ composite showed an improved photocatalytic activity and stability for the degradation of rhodamine B (RhB), a model organic dye, under visible light irradiation. The decomposition ratio of RhB reached 87.1% after exposure to visible light for 100 min, corresponding to a reaction rate constant that was 4.8 and 15.9 times that of carbon cloth-supported Ag_3_PO_4_ or ZnO alone. The enhanced performance of the composite can be attributed to the effectively inhibited recombination of photoinduced electron–hole pairs by the S-scheme heterojunction. The carbon fibers further promoted the transfer of charges. Moreover, the carbon cloth-supported ZnO@Ag_3_PO_4_ can be easily separated from the solution and repeatedly used, demonstrating a fair recyclability and potential in practical applications.

## 1. Introduction

With the rapid development of the textile and dyeing industry, large quantities of organic dye-laden wastewater have been discharged into the natural waters, causing a range of environmental problems. Many of the organic dyes are toxic to human health or aquatic life [1,2]. Over the past decades, various destructive and nondestructive techniques have been explored for removing organic dyes from wastewater, including adsorption, filtration, biodegradation, advanced oxidation processes, photocatalysis, etc. [3,4,5,6]. Amongst these approaches, semiconductor-based photocatalysis can degrade and detoxify organic pollutants, including dyes, utilizing inexhaustible solar energy, which is expected to solve the problems of increasingly serious water pollution by organic dyes [7,8,9,10].

Zinc oxide (ZnO) is a versatile semiconductor photocatalysts with high ultraviolet (UV) shielding and refractive index, high electron mobility, and strong luminescence at room temperature [11], and ZnO-based photocatalysis has been widely explored for removing wastewater pollutants, including organic dyes [12,13,14]. However, as a typical broad-bandgap semiconductor, ZnO can be mainly excited by UV light, resulting in a low utilization rate of the visible light fraction of solar radiation, which, together with its vulnerability to corrosion under both irradiated and dark conditions [15,16], hinders the large-scale application of pure ZnO-based materials in the field of environmental photocatalysis [17]. The coupling of two band-matched semiconductors to form a heterojunction is an effective way to reduce the recombination of photoelectron–hole pairs and improve the performance of photocatalytic materials [18,19]. Moreover, the coating of a material less prone to corrosion onto the surface of ZnO can dramatically enhance the stability of ZnO-based photocatalysts [15,20]. Since Ye et al. reported the use of silver phosphate (Ag_3_PO_4_) in water oxidization and organic contaminant decomposition, Ag_3_PO_4_, a visible light-responsive semiconductor, has been widely considered as a promising material for solar photocatalysis [21,22,23]. Despite its low solubility in water, however, we have previously observed that, during extended photocatalytic tests, photoelectrons can induce the precipitation of metallic Ag films on the surface of Ag_3_PO_4_, which shield light and lead to the deactivation of Ag_3_PO_4_ [24]. It has been demonstrated that the combination of ZnO with Ag_3_PO_4_ could improve the photocatalytic activity of ZnO/Ag_3_PO_4_ [25,26,27], though an improvement in performance stability is less straightforward, with contradicting results reported [25,28].

Up to now, a majority of the reported photocatalysts exist in the state of powder, which causes difficulty in separation and recycling when it comes to practical applications [29,30], highlighting the need for preparing immobilized photocatalysts with both high activity and recyclability [31]. Carbon cloth has unique physical and chemical properties such as a large specific surface area, excellent electrical conductivity, high strength, good thermal stability, and good corrosion resistance, which has been widely used in supercapacitors, lithium–ion batteries, electrocatalysis, microwave absorption, and other fields [32,33,34]. It was reported in our previous work that using carbon cloth as the catalyst support can effectively improve the photocatalytic performance and recyclability of the photocatalyst [35]. Thus, it is hypothesized that loading composites of ZnO and Ag_3_PO_4_ with well-designed structures onto carbon cloth may yield a recyclable photocatalyst with high activity.

In this work, we reported the synthesis of a hybrid ZnO@Ag_3_PO_4_ core–shell structure grown on carbon cloth via a calcination treatment followed by a two-step solution route (including a solvothermal treatment and subsequent impregnation–deposition) and evaluated its performance for the photocatalytic degradation of rhodamine B (RhB), a widely used organic dye known to be toxic to both humans and aquatic life [1,36]. The ZnO@Ag_3_PO_4_ heterostructure is conductive to photocatalytic activity improvement under visible light irradiation, whereas the carbon fibers, as high-speed electron transfer channels, are also beneficial to charge a separation. As a result, the carbon cloth-supported ZnO@Ag_3_PO_4_ composite material exhibits a dramatic visible light photocatalytic activity compared with that of carbon cloth-supported ZnO and Ag_3_PO_4_. Additionally, the free-standing carbon cloth-supported ZnO@Ag_3_PO_4_ can be easily withdrawn from a simulated wastewater solution and reused, which makes it a recyclable photocatalyst with potential for practical applications.

## 2. Materials and Methods

**Figure 1:**
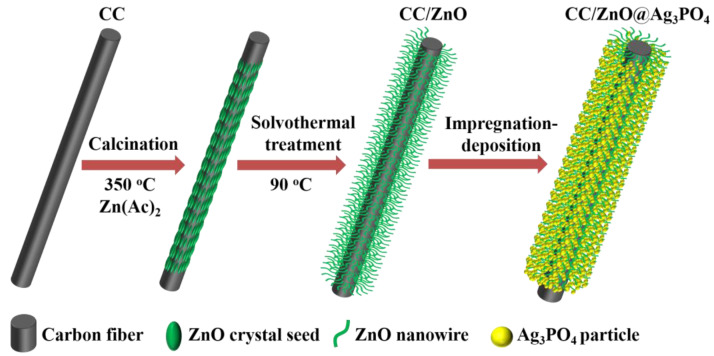
Schematic illustration for the synthesis procedure of the carbon cloth-supported ZnO and ZnO@Ag_3_PO_4_ composite.

The procedures for synthesizing the materials are illustrated in Figure 1.

### 2.1. Deposition of ZnO Crystal Seeds on Carbon Cloth

The carbon cloth (WOS: 1009-17070602, average thickness: 0.33 mm, and nominal basic weight: 115.0 g/m^2^) with dimensions of 2 cm × 2 cm was successively dipped into acetone, anhydrous ethanol, and deionized water for 20 min in ultrasonic bath to remove the surface impurities. Then, the carbon cloth was soaked in nitric acid (65%) for 24 h to improve the surface hydrophilicity. After that, the carbon cloth was cleaned with deionized water several times and dried in an oven at 60 °C for 12 h.

ZnO crystal seeds were deposited on the carbon cloth by the calcination method. Specifically, 0.18 mmol of anhydrous zinc acetate was completely dissolved in 30 mL of anhydrous ethanol under continuous magnetic stirring. Afterwards, the mixed solution was slowly dripped onto the pretreated carbon cloth, and the obtained wet carbon cloth was further heated in a muffle furnace at 350 °C for 20 min in air. After cooling to room temperature, white ZnO crystal seeds could be found to adhere to the surfaces of carbon fibers.

### 2.2. Growth of ZnO Nanowires on Carbon Cloth

ZnO nanowires were grown on carbon cloth by a simple solvothermal method. Firstly, 3 mmol of zinc nitrate hexahydrate (Zn (NO_3_)_2_·6H_2_O) and 2 mmol of hexamethylenetetramine (HMTA) were dissolved in a certain volume of mixed solution containing aqueous ammonia (3 mL) and deionized water (70 mL), followed by being poured into a 100 mL autoclave with Teflon liner. Subsequently, the carbon cloth with ZnO crystal seeds was vertically soaked into the solution, and then, the autoclave was sealed and heated at 90 °C for 24 h. After the reaction, the autoclave naturally cooled down to room temperature. Finally, the carbon cloth with ZnO nanowires (denoted as CC/ZnO) was removed from the autoclave and washed with deionized water and ethanol several times and dried in an oven at 60 °C overnight.

### 2.3. Preparation of Carbon Cloth-Supported ZnO@Ag_3_PO_4_ Composite

The carbon cloth-supported ZnO@Ag_3_PO_4_ composite was prepared by a simple impregnation–deposition method. The whole experiment process was carried out in dark conditions to avoid undesired photo corrosion. A certain amount of AgNO_3_ and (NH_4_)_2_HPO_4_ were separately dissolved in deionized water to form solutions with concentrations of 0.5 and 0.17 mol/L, respectively. Then, the as-prepared carbon cloth-supported ZnO was soaked in AgNO_3_ solution for 6 h. After that, the wet AgNO_3_-infiltrated CC/ZnO was taken out and immersed in (NH_4_)_2_HPO_4_ solution for 1 min and then was again dipped back into the AgNO_3_ solution. This soaking process was repeated 20 times. Finally, the obtained carbon cloth-supported ZnO@Ag_3_PO_4_ composite (denoted as CC/ZnO@Ag_3_PO_4_) was washed with deionized water and dried at 60 °C for 12 h. For comparison, carbon cloth-supported Ag_3_PO_4_ (denoted as CC/Ag_3_PO_4_) was also prepared by directly soaking pure carbon cloth into the precursor solutions in the same way as described above.

### 2.4. Characterization

The morphological observation of the carbon cloth-supported photocatalysts was accomplished on a SU8010 field emission scanning electron microscope (FESEM, Hitachi, Japan) and a JEM2010F transmission electron microscope (TEM, JEOL, Japan). The crystal structure and phase composition of the samples were analyzed by a D/MAX-Ultima IV X-ray diffractometer (XRD, Rigaku, Japan) using Cu Kα radiation with a scanning rate of 8°/min. X-ray photoelectron spectroscopy (XPS) was measured on an EscaLab 250Xi electron spectrometer (Thermo, Waltham, MA, USA) excited by using Al Kα radiation. UV–Vis diffuse reflectance spectra (DRS) of the samples were recorded on a UV9000 UV–Vis spectrophotometer (Metash, Shanghai, China), with BaSO_4_ used as a reflectance standard.

### 2.5. Photocatalytic Activity Measurement

The photocatalytic activities of the carbon cloth-supported photocatalysts were evaluated by the degradation of RhB (1.0 × 10^−5^ mol/L) as the target organic pollutant at room temperature, and a LED lamp with a fixed wavelength of 420 nm was used as the visible light source to trigger the reaction. To be more specific, a piece of carbon cloth-supported photocatalyst (2 cm × 2 cm) was vertically placed into a beaker containing 60 mL of RhB solution. Before turning on the LED light, the catalytic reaction system was magnetically stirred in dark for 30 min to reach adsorption equilibrium. During the process of light irradiation, aliquots of the supernatant were removed with a disposable dropper at regular time intervals and transfused into a colorimeter cell. The absorbance of the solution was measured by a UV–visible spectrophotometer, and the activities of the photocatalysts were estimated by comparing the apparent reaction rate constant, which can be calculated by ln(*C*_0_/*C_t_*) = *kt*. In this equation, *C*_0_ and *C_t_* represent the concentration of RhB solution initially at the adsorption equilibrium (before the light was turned on) and at the reaction time of *t*, respectively, where *k* represents the reaction rate constant, and *t* represents the irradiation time.

## 3. Results and Discussion

### 3.1. Phase Structure

**Figure 2:**
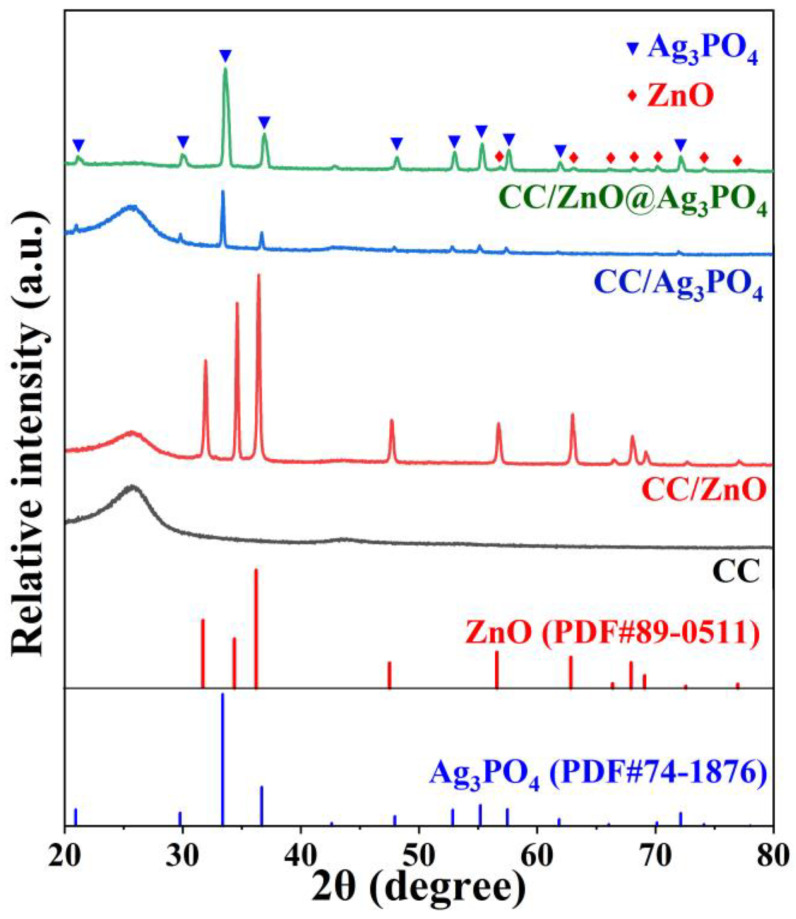
XRD patterns of the CC, CC/ZnO, CC/Ag_3_PO_4_, and CC/ZnO@Ag_3_PO_4_ composites.

The crystal structure and phase composition of the samples were characterized by XRD, and the related patterns are shown in Figure 2. The pure carbon cloth (CC) exhibits a relatively wide diffraction peak at 26°, corresponding to the (002) plane of the carbon fiber [37]. In the case of CC/ZnO, besides the weak peak of carbon cloth, many new diffraction peaks (labeled with ♦) appear in the XRD pattern, including three strong peaks at 31.7°, 34.4°, and 36.3°, which can be assigned to the (100), (002), and (101) planes of the hexagonal ZnO (JCPDS card No. 89-0510), respectively [38]. This indicates that ZnO has successfully grown on the carbon cloth. The XRD pattern of CC/Ag_3_PO_4_ shows diffraction peaks corresponding to both carbon fiber and Ag_3_PO_4_ (JCPDS card No. 74-1876, labeled with ▼) [39]. As for the CC/ZnO@Ag_3_PO_4_ composite, its XRD pattern displays strong diffraction peaks of Ag_3_PO_4_ and extremely weak diffraction peaks of ZnO, which is probably due to the full coverage of ZnO by Ag_3_PO_4_. Meanwhile, the characteristic peak of carbon fiber at 26° is practically unobservable, mainly attributed to the fact that the surface of the carbon fibers was entirely coated by ZnO and Ag_3_PO_4_. In addition, the peak intensity of Ag_3_PO_4_ in CC/ZnO@Ag_3_PO_4_ is stronger than that in CC/Ag_3_PO_4_. It can be inferred that more Ag_3_PO_4_ was deposited on the surface of CC/ZnO than directly on the pure carbon cloth.

### 3.2. Morphology

**Figure 3:**
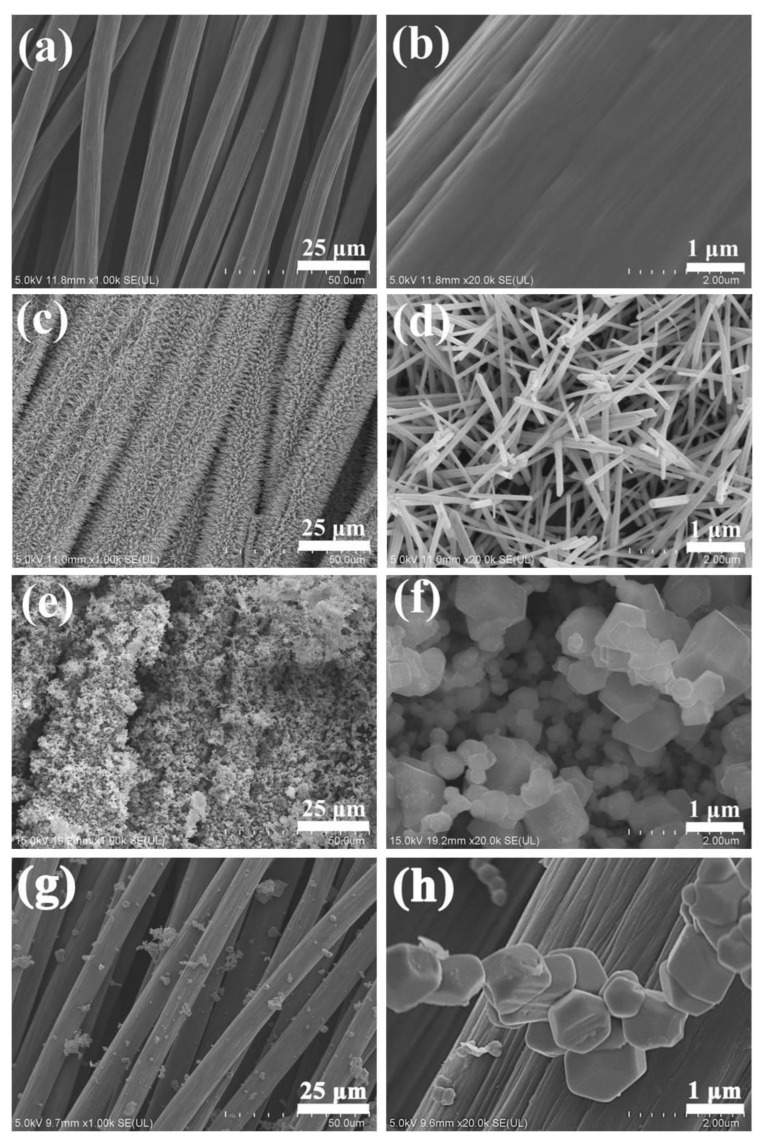
SEM images of CC (**a**,**b**), CC/ZnO (**c**,**d**), CC/ZnO@Ag_3_PO_4_ (**e**,**f**), and CC/Ag_3_PO_4_ (**g**,**h**).

The morphology of the bare carbon cloth, CC/ZnO, CC/Ag_3_PO_4_, and CC/ZnO@Ag_3_PO_4_ was investigated by SEM (Figure 3). The microstructure of the bare carbon cloth consisted of carbon fibers with an average diameter of about 10 μm (Figure 3a). A high-magnification SEM image showed that the surface of the carbon fiber was relatively smooth, with wrinkles parallel to the direction of the fibers (Figure 3b). After solvothermal treatment in the precursor solution of ZnO, the carbon fibers became brushy, and many nanowires were found to grow evenly on the surface (Figure 3c). More detailed observation revealed that these ZnO nanowires were crisscross-distributed on the carbon fibers with diameters of 60–160 nm and lengths of more than 2 μm (Figure 3d). The ZnO nanowires with a crisscross structure tended to have high specific surface areas that were available for the deposition of more catalyst particles or adsorption of target molecules. As expected, the surface of CC/ZnO was completely covered by a large number of particles after the deposition of Ag_3_PO_4_ (Figure 3e). Further observation of the CC/ZnO@Ag_3_PO_4_ composite showed that these Ag_3_PO_4_ particles have a polyhedral shape with sizes of 300 nm to 1 μm (Figure 3f). Meanwhile, the ZnO nanowires can be hardly seen in Figure 3f, probably because they were entirely encased by the Ag_3_PO_4_ particles. In contrast, it is evident from Figure 3g,h that only a small quantity of Ag_3_PO_4_ particles are sparsely distributed on the surface of the carbon fibers in CC/Ag_3_PO_4_, owing to the relatively smooth surfaces of carbon fibers. The mass loading of ZnO@Ag_3_PO_4_ and pure Ag_3_PO_4_ on the carbon cloth was 7.8 × 10^−3^ and 1.8 ×10^−3^ g/cm^2^, respectively. The above results are in good correspondence with the XRD analysis.

**Figure 4:**
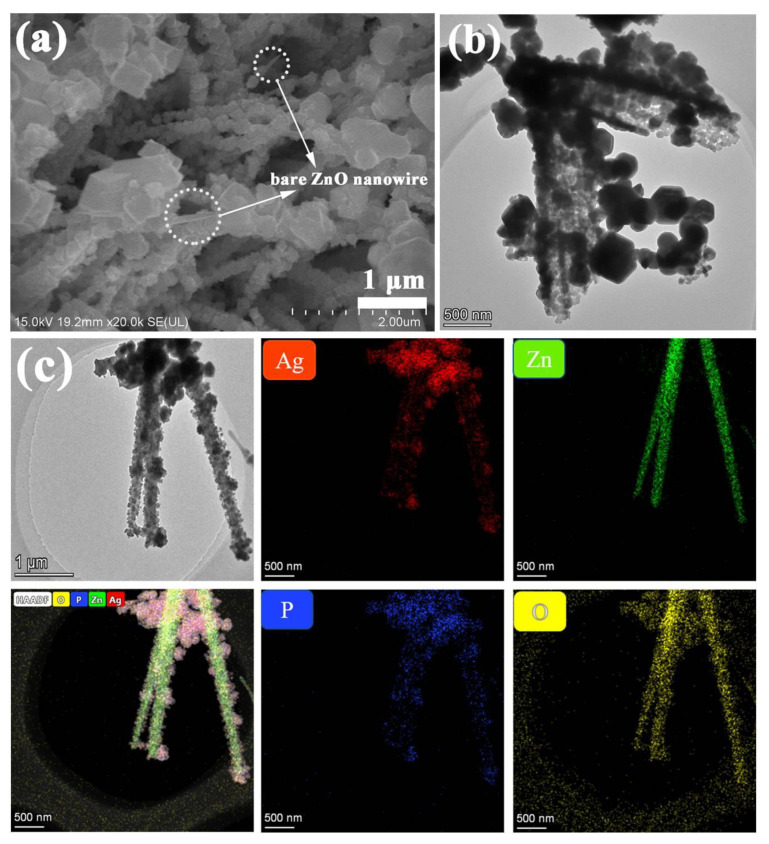
SEM image (**a**), TEM image, (**b**) and corresponding elemental mappings (**c**) of the CC/ZnO@Ag_3_PO_4_ composite, which show distribution of the Ag, Zn, P, and O elements.

In order to further investigate the presence of ZnO nanowires in the CC/ZnO@Ag_3_PO_4_ composite, a control experiment was carried out by shortening the repeated soaking times of CC/ZnO in AgNO_3_ and (NH_4_)_2_HPO_4_ solutions to 15 times, and the SEM image of the as-obtained CC/ZnO@Ag_3_PO_4_ composite is shown in Figure 4a. Obviously, many nanoparticles aggregated on the surfaces of ZnO nanowires (marked by the dotted circle). The diameter of these ZnO@Ag_3_PO_4_ nanowires was about 200 nm. In addition, the ZnO@Ag_3_PO_4_ nanowires were scraped off from the surfaces of carbon fibers for TEM characterization to further confirm the existence of ZnO nanowires. As shown in Figure 4b, the nanowires (dark) were completely covered by many nanoparticles (light) with sizes of about 75 nm, forming a core–shell structure. Additionally, bigger polyhedral particles were found to be adjacent to these nanoparticles. The TEM image is well consistent with the SEM observations in Figure 4a. Furthermore, the elemental mappings of the Ag, Zn, P, and O elements also support the core–shell structure of the CC/ZnO@Ag_3_PO_4_ composite (Figure 4c). The Zn element only exists in the core nanowires, while the Ag, P, and O elements are distributed near the ZnO nanowires but cover a larger extent. Such tight contact between ZnO and Ag_3_PO_4_ is conductive to the transfer of charge carriers.

### 3.3. Surface Chemistry

**Figure 5:**
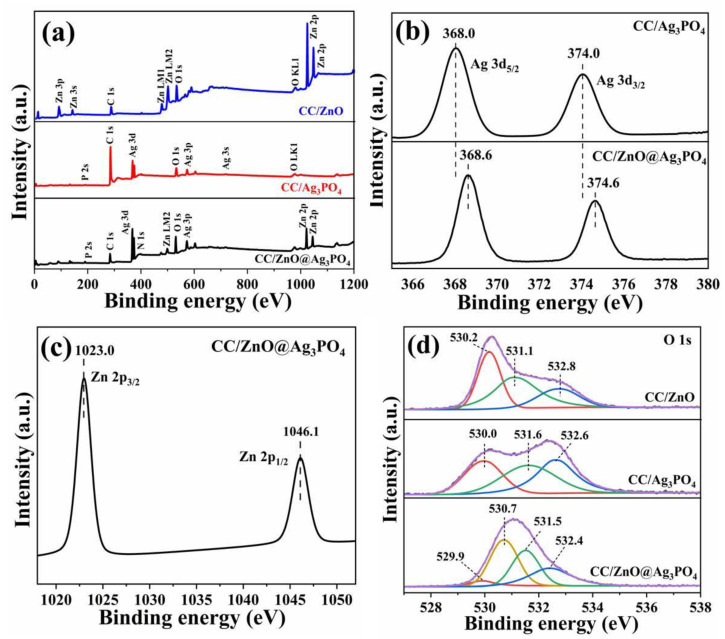
XPS survey spectrum (**a**) and high-resolution XPS spectra of Ag 3d (**b**), Zn 2p (**c**), O 1s (**d**) of CC/ZnO, CC/Ag_3_PO_4_, and the CC/ZnO@Ag_3_PO_4_ composite.

The surface composition and chemical states of CC/ZnO, CC/Ag_3_PO_4_, and CC/ZnO@Ag_3_PO_4_ were further determined by XPS, and the obtained spectra are shown in Figure 5. The full-scanned spectrum of CC/ZnO@Ag_3_PO_4_ indicates the presence of the Zn, Ag, O, P, and C elements (Figure 5a), which originated from the ternary composites containing carbon fiber, ZnO, and Ag_3_PO_4_. A close look at the full spectra reveals that the C 1s peak intensity of CC/Ag_3_PO_4_ is much stronger than that of CC/ZnO and CC/ZnO@Ag_3_PO_4_, owing to the low loading of Ag_3_PO_4_ on the pure carbon cloth, which results in a larger fraction of surface-exposed carbon fibers. This result is in good agreement with the XRD analysis. The high-resolution XPS spectrum of Ag 3d in CC/Ag_3_PO_4_ centered at 374.0 and 368.0 eV (Figure 5b), corresponding to the Ag 3d_3/2_ and Ag 3d_5/2_ core levels of Ag^+^, respectively [34]. Meanwhile, the Ag 3d peaks in CC/ZnO@Ag_3_PO_4_ exhibited a positive shift by 0.6 eV compared with those for CC/Ag_3_PO_4_, reflecting an increase in the electron density around the Ag ions (corresponding to a lower formal charge) [40,41], which likely came from the transfer of electrons from ZnO to Ag_3_PO_4_. The characteristic peaks of Zn 2p appeared at 1023.0 and 1046.1 eV (Figure 5c), which correspond to Zn 2p_3/2_ and Zn 2p_1/2_, indicating the existence of Zn^2+^ from ZnO [42]. The O 1s peak of CC/ZnO could be fitted into three peaks, as shown in Figure 5d. The dominant peak at 530.2 eV was from the Zn–O bonding of ZnO, and the other two peaks at 531.1 and 532.8 eV were associated with dissociatively adsorbed water (Zn–OH) and physically adsorbed H_2_O molecules, respectively [43,44]. For CC/Ag_3_PO_4_, the O 1s XPS spectrum could also be deconvoluted into three peaks at 530.0, 531.6, and 532.6 eV, which are related to the lattice oxygen of Ag_3_PO_4_, chemisorbed oxygen of surface –OH group, and physically adsorbed H_2_O molecules [45,46,47]. The high-resolution XPS spectrum of O 1s in CC/ZnO@Ag_3_PO_4_ could be disintegrated into four peaks. The two peaks at lower binding energy (529.9 and 530.7 eV) are assigned to the lattice oxygen of Ag_3_PO_4_ and Zn–O bonding of ZnO [48,49], respectively. The other two peaks at higher binding energy (531.5 and 532.4 eV) are related to the surface –OH group and H_2_O molecules absorbed on the surface of the CC/ZnO@Ag_3_PO_4_ composite [50]. The slight shift of O 1s peaks related to Zn–O and Ag–O in the CC/ZnO@Ag_3_PO_4_ composite was observed, compared with O 1s peaks in CC/ZnO and CC/Ag_3_PO_4_, implying the formation of chemical bonding between ZnO and Ag_3_PO_4_. The above XPS results further confirmed the successful synthesis of a CC/ZnO@Ag_3_PO_4_ composite photocatalyst.

### 3.4. Optical Absorption Property

**Figure 6:**
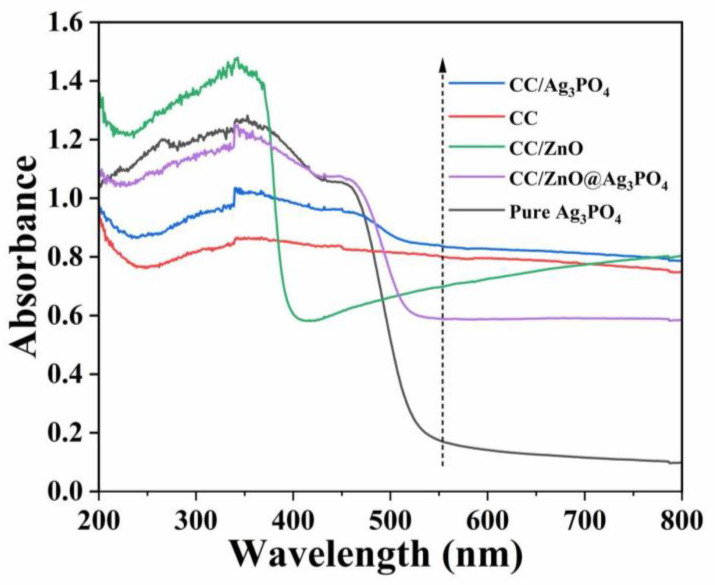
UV–Vis diffuse reflectance spectra of the as-prepared samples.

The UV–Visible DRS test was carried out to explore the optical absorption of CC, CC/ZnO, CC/Ag_3_PO_4_, CC/ZnO@Ag_3_PO_4_, and pure Ag_3_PO_4_ powder, and the results are shown in Figure 6. It can be found that the dark grey CC has a strong light absorption within the entire UV–visible light region examined. The CC/ZnO exhibited strong absorption in the ultraviolet light region of 200–400 nm, and its relatively strong absorption in the visible light region was brought about by the carbon cloth. The intrinsic absorption edge of pure Ag_3_PO_4_ powder is about 536.5 nm, corresponding to a bandgap energy of 2.31 eV. The CC/ZnO@Ag_3_PO_4_ also displayed a similar light absorption curve to that of pure Ag_3_PO_4_, except for an enhanced absorption in the 470–800 nm region, possibly because the unique structure of the ZnO nanowires grown on carbon fibers enhanced light scattering. This can also be verified from the relatively strong absorption of CC/ZnO in the visible light region. In addition, the light absorbance of CC/Ag_3_PO_4_ is higher than that of CC/ZnO@Ag_3_PO_4_, which can be attributed to the fact that the quantity of Ag_3_PO_4_ particles directly deposited on the surface of the carbon cloth was much less than that deposited on CC/ZnO, leading to more exposed carbon fibers in CC/Ag_3_PO_4_ than in CC/ZnO@Ag_3_PO_4_. It is well known that the dark grey carbon fibers can almost absorb all the light. The above results also match well with the XRD and SEM analyses.

### 3.5. Photocatalytic Dye Removal Performance

**Figure 7:**
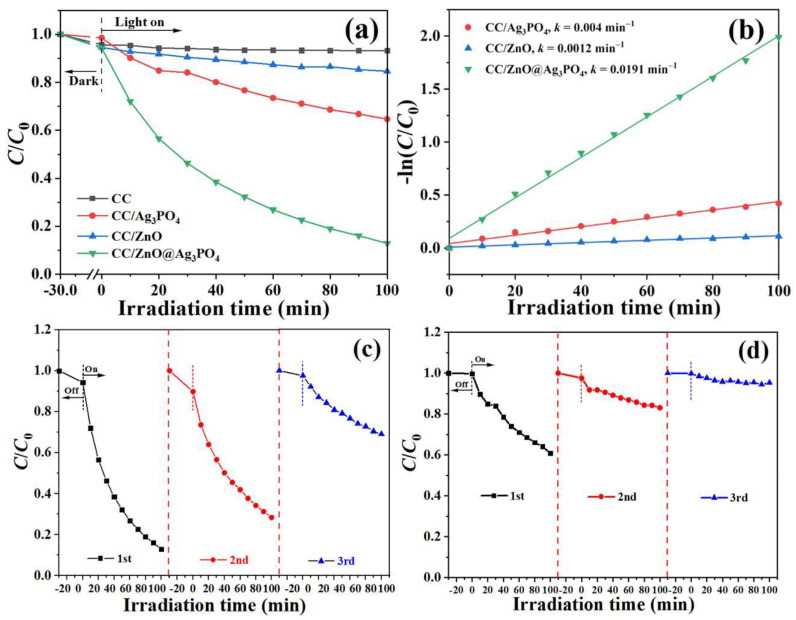
Comparison of the photocatalytic activities (**a**) and apparent reaction rate constants (**b**) of CC, CC/ZnO, CC/ZnO@Ag_3_PO_4_, and CC/Ag_3_PO_4_ for the degradation of RhB under visible light irradiation, and photocatalytic recyclability of the CC/ZnO@Ag_3_PO_4_ composite (**c**) and CC/Ag_3_PO_4_ (**d**).

The photocatalytic activities of the prepared samples for the removal of RhB from an aqueous solution were performed under visible light from LED lamp irradiation. As can be seen from Figure 7a, only a small amount of RhB was adsorbed for all samples in the dark conditions. The CC/ZnO sample exhibited weak photocatalytic activity, with only about 15.4% removal achieved after 100 min of light illumination. The CC/Ag_3_PO_4_ sample also exhibited a modest photocatalytic performance, with 35.3% RhB removal. By contrast, 87.1% of RhB was degraded by the CC/ZnO@Ag_3_PO_4_ composite, which showed the highest photocatalytic activity with a reaction rate constant of 0.0191 min^−1^, exceeding that of CC/Ag_3_PO_4_ by 4.8 times and that of CC/ZnO by a factor of 15.9 (Figure 7b). The stability and reusability are essential to the practical application of the photocatalysts. Herein, three cycles of photocatalytic experiments were carried out to investigate the photocatalytic stability of CC/ZnO@Ag_3_PO_4_ and CC/Ag_3_PO_4_. The RhB removal ratio for the CC/ZnO@Ag_3_PO_4_ composite was 30.9% after three cycle tests (Figure 7c), showing a 64.5% activity loss compared with its performance in the first test. However, only 4.7% of the decomposition ratio of RhB remained for CC/Ag_3_PO_4_ after three cycles of photocatalytic degradation tests (Figure 7d), which exhibited an 88.0% activity loss compared with itself. These results indicated that the heterostructure formed between ZnO and Ag_3_PO_4_ is beneficial to the improvement of photocatalytic activity and stability of the CC/ZnO@Ag_3_PO_4_ composite.

## 4. Possible Photocatalytic Mechanism

**Figure 8:**
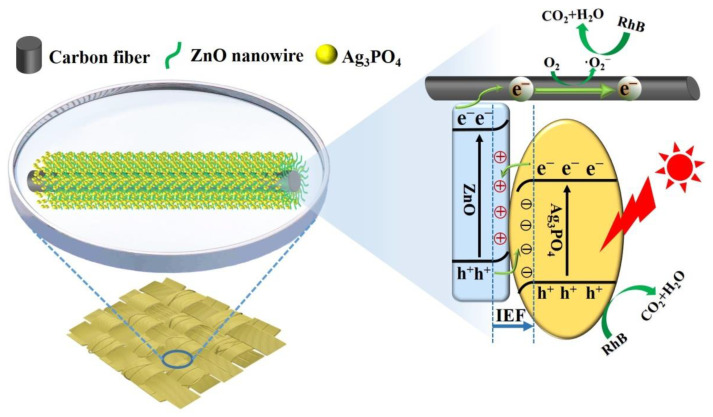
Schematic diagram for the improved charge separation of the CC/ZnO@Ag_3_PO_4_ composite.

On the basis of the above results and the literature, a possible charge separation and transfer mechanism for the enhanced activity and stability of the CC/ZnO@Ag_3_PO_4_ composite was proposed (Figure 8). According to the literature, ZnO possesses a more negative valence band (VB) and conduction band (CB) edge than that of Ag_3_PO_4_ [51]. When they come into contact with each other, electrons transfer from ZnO to Ag_3_PO_4_, and an internal electric field (IEF) is created at the interface of ZnO and Ag_3_PO_4_ [52]. The direction of the IEF points from ZnO to Ag_3_PO_4_, as marked by the arrow in Figure 8. Under visible light excitation, ZnO shows weak photocatalytic activity, probably due to its intrinsic defect in the crystal and inefficient light absorption [53]. Simultaneously, both ZnO and Ag_3_PO_4_ can be excited, and the electrons in the VB leap into the CB, while the positively charged holes (h^+^) are left in the VB. Subsequently, the photogenerated electrons in the CB of Ag_3_PO_4_ shift towards ZnO, as driven by the interfacial IEF, and recombine with the h^+^ in the VB of ZnO. Apparently, an S-scheme mechanism applies well to the heterojunction between Ag_3_PO_4_ and ZnO, and the transfer of electrons and holes in the IEF region follows a slide-like pathway. Benefitting from this ZnO@Ag_3_PO_4_ S-scheme heterojunction, the photogenerated charges efficiently separate. Consequently, the holes are enriched in the VB of Ag_3_PO_4_, while the electrons are enriched in the CB of ZnO. It has been reported that the h^+^ and superoxide anion radicals (·O_2_^−^) are the main active species in the ZnO/Ag_3_PO_4_ reaction system, according to the free radical capture experiment [54,55]. The holes reserving high oxidation ability in the VB of Ag_3_PO_4_ can directly participate in the degradation of RhB. In addition, it is well known that the carbon fibers with a one-dimensional linear structure possess excellent electrical conductivity, which rapidly receive electrons from the CB of ZnO. Then, the dissolved oxygen molecules are captured by the electrons enriched in the carbon fibers and reduced into ·O_2_^−^ radicals, which further degrade RhB molecules into CO_2_, H_2_O, and small molecular compounds [35]. Moreover, the ZnO nanowires directly grown on the conductive carbon fibers ensure good adhesion of the ZnO@Ag_3_PO_4_ core–shell structure with the carbon cloth substrate.

## 5. Conclusions

In summary, the carbon cloth-supported ZnO@Ag_3_PO_4_ composite with a core–shell structure was successfully prepared by a two-step process including a solvothermal method and a succeeding impregnation–deposition method. The as-prepared CC/ZnO@Ag_3_PO_4_ displayed an enhanced photocatalytic activity for degrading RhB under visible light irradiation compared with that of CC/ZnO and CC/Ag_3_PO_4_, mainly attributed to the synergistic effect of the carbon cloth, ZnO, and Ag_3_PO_4_. The construction of a S-scheme heterojunction between ZnO and Ag_3_PO_4_ was mainly responsible for the enhanced activity of the composite, which suppressed the recombination of the charge carriers within ZnO or Ag_3_PO_4_ itself by transferring electrons from the CB of Ag_3_PO_4_ to the VB of ZnO through the internal electric field. Additionally, carbon fibers further accelerated the transmission of electrons; therefore, the charge separation efficiency was further improved. The carbon cloth-supported ZnO@Ag_3_PO_4_ can be easily separated from the solution and repeatedly used, demonstrating a fair recyclability and potential in practical applications. Nevertheless, the decreased activity of CC/ZnO@Ag_3_PO_4_ after many cycles of photocatalytic tests still needs to be further explored.

## Data Availability

All the data are available within the manuscript.
